# The development and validity of the Early Learning Tool for children 0–3-year-old in rural Pakistan

**DOI:** 10.7189/jogh.14.04241

**Published:** 2024-11-22

**Authors:** Elizabeth Hentschel, Saima Siyal, Alya Al Sager, Dana C McCoy, Aisha K Yousafzai

**Affiliations:** 1 Harvard T.H. Chan School of Public Health, Department of Global Health and Population, Boston, Massachusetts, USA; 2 Development and Research for Children in Early Adolescent Years of Life (DREAM) Non-Governmental Organization, Naushahro Feroze, Sindh, Pakistan; 3 Harvard Graduate School of Education, Cambridge, Massachusetts, USA

## Abstract

**Background:**

Research has demonstrated the critical role that early learning experiences play in shaping children’s cognitive, social, and emotional development. Nevertheless, tools for assessing children’s exposure to early learning experiences remain scarce. This paper describes the initial validation of the Early Learning (EL) tool, which captures the levels of stimulation with playthings and people available to children 0–3-year-old in low-resource, international settings.

**Methods:**

We randomly sampled 200 mothers of children under 3-year-old in rural Sindh, Pakistan. We collected data on sociodemographic information, early learning, responsive caregiving, and child development. Psychometric analyses assessing the structural validity with confirmatory factor analysis and item response theory, criterion validity with Pearson correlation coefficients, and predictive validity with Ordinary Least Squares linear regression were conducted.

**Findings:**

We found that the EL tool is valid in this setting, capturing two factors of early learning: stimulation with playthings and stimulation with people. Stimulation with playthings and people were strongly and positively correlated with responsive caregiving, maternal education, and wealth, indicating criterion validity. Stimulation with playthings and people were also strongly associated with child development, measured by the Bayleys Scales of Infant Development.

**Conclusions:**

The EL tool is a promising instrument for measuring early learning in low- and middle-income countries, and its use can lead to more effective and inclusive monitoring and development of early learning initiatives.

An expanding body of evidence has pointed to the unique importance of the earliest years of life (0–3 years) as a period of rapid growth in children’s cognitive, social, and emotional development [1,2]. During this formative period, children develop essential skills in response to environmental stimuli, particularly early learning (EL) experiences, which include a range of stimulating activities (e.g. incidental learning through free play, adult-guided instruction), enhancing child development and facilitating knowledge acquisition [3].

The global emphasis on nurturing care, as outlined by UNICEF, the World Health Organization (WHO), and the World Bank’s Nurturing Care Framework (NCF) has brought EL to the forefront of international child development agendas [4]. The NCF highlights a variety of early learning activities that are critical for children’s development across diverse cultural contexts, including playing with household objects, talking, singing, imitating, and participating in simple games. However, despite the recognised importance of these activities, there remains a significant gap in tools that can accurately measure EL experiences, particularly in low- and middle-income countries (LMICs), thereby limiting our ability to evaluate and enhance these early learning environments [5].

Existing tools, such as the Family Care Indicators (FCI) [6] and the Home Observation Measurement of the Environment-Infant Toddler (HOME-IT) [7] are commonly used to assess early learning globally. Although the FCI, effective for assessing the impact of parenting programmes on play, has been adapted in various settings, including Bangladesh [8], and incorporated into UNICEF’s Multiple Indicator Cluster Survey (MICS), it is more suited for children over three years old, with items such as ‘things for learning shapes and colors’ and ‘things for drawing and writing’ being less relevant for younger children [9]. Further, the FCI was developed to measure home stimulation, not early learning explicitly. The HOME-IT provides a detailed evaluation of the home environment's impact on child development, assessing aspects such as emotional and verbal responsivity and learning materials availability [10] but was not developed to assess early learning as a distinct concept. While the HOME-IT has been adapted for use in various countries [10-12]. it is challenging to administer and includes many observational items, which reduces its practical applicability in low-resource settings [13]. Moreover, studies have shown that in LMICs, the HOME-IT’s subscales often load onto one factor in LMICs [13], complicating the interpretation of the independent impact of specific dimensions of EL.

Given these limitations, there is a pressing need for more targeted, culturally adaptable, and easy-to-administer tools that accurately capture early learning experiences in diverse settings. The EL tool was developed to address this gap by focusing on stimulation through playthings and social interaction. Building on the FCI and HOME-IT, the EL tool underwent a rigorous development process, including the creation of a conceptual framework, item set creation, and iterative qualitative and quantitative testing [9]. The final tool features 14 items – seven capturing stimulation with playthings and seven with people in the previous 24 hours [9]. The detailed process for developing this tool is outlined in [9]. The EL tool’s unique contribution lies in its specific design for children under three years old, its applicability across different cultural settings, its ease of use in low-resource environments (requires minimal training and can be administered in under five minutes) and its explicit focus on the concept of early learning, as defined by the NCF. The EL tool has previously been used in Pakistan and was found to be the strongest predictor of child development under three in a nationally representative survey [14].

The overall aim of the current study is to provide evidence regarding the psychometric properties of the EL tool in families with children under three years old in the Naushahro Feroze District of Sindh, Pakistan. The specific objectives of this study are to

1) establish construct validity through confirmatory factor analysis

2) assess criterion validity by examining correlations with established measures of early stimulation (the HOME-IT) and responsive caregiving (RC tool)

3) evaluate predictive validity concerning child development outcomes measured by the Bayley Scales of Infant Development, 4th edition (Bayley-4).

We hypothesise that the EL tool will demonstrate strong validity, effectively capturing critical aspects of early learning essential for the development of young children in rural Pakistan. This validation study will contribute a feasible and valid instrument to the field of early childhood development, with potential applications in other LMIC settings.

## METHODS

### Study setting and sample

We collected data from a sample of 200 randomly selected households that had children under three years of age, across six villages within the rural district of Naushahro Feroze, Sindh, Pakistan. Naushahro Feroze is primarily recognised for its agricultural livelihood. A significant portion of the population, 25% across all genders and ages, is burdened with multidimensional poverty [15] and regional statistics indicate that only 47.5% of 3–4-year-old children in Sindh are developmentally on track, a figure substantially lower than the 75.1% average seen in LMICs [15]. A list of households was provided by the Naushahro Feroze Lady Health Workers. The 200 randomly selected households were stratified based on four child age brackets: 3–6 months, 7–12 months, 13–24 months, and 25–36 months. Within each stratum, approximately 50 households were chosen at random, resulting in 48 from the first age group, 48 from the second, 53 from the third, and 51 from the final age group.

### Field testing and piloting

The initial establishment and testing phase of instruments took place from 10 July 2021 to 30 July 2021, and field testing, piloting, and data collection were conducted between August 2021 until October 2021. Qualitative interviews were first conducted among a purposive sample of 20 caregivers with children aged 0–3 years in Naushahro Feroze. The objective was to gain a deeper understanding of the implementation and comprehension of responsive care and early learning within the rural Pakistan context. The qualitative findings indicated that caregivers frequently engaged in responsive and stimulating interactions with their children [16]. Following these interviews, a training programme was organised to ensure the proficient application of the EL tool. Post-training, a nine-day field evaluation was conducted to preliminarily test the EL tool, along with the Bayley Scale of Infant Development, 4th edition (Bayley-4), Home Observation for Measurement of the Environment-Infant Toddler (HOME-IT), and the Responsive Caregiving Indicator Tool (RC Tool) [17], and the demographic questionnaire. All tools were translated into Sindhi and had previously been adapted and used in Sindh, Pakistan [12,18-22]. Upon completion of the field evaluation, a two-day operational pilot was carried out to ensure the efficacy and accuracy of all implemented measures and methodologies.

### Data collection

Data collection spanned from July to October 2021 four research assistants who all lived and were from Naushahro Feroze, each had master’s degrees, and each over ten years of ECD-related research experience. All data was collected in Sindhi. at the home of the interviewee and confidentiality was maintained. Each interview began with a brief introduction of the study. Research assistants then received informed consent from all participants verbally and with their signatures or thumbprints. All forms were read aloud by the research assistant in Sindhi. Participants were given the opportunity to ask questions and to withdraw at any point. Participants were not financially compensated, but received soap and a small toy for participating in the interview. The study was evaluated by the Institutional Review Boards of each site, Institutional Review Board at the Harvard T.H. Chan School of Public Health IRB21-0579 and National Institutes of Health, Health Research Institute National Bioethics Committee (NBC) Ref: No.4-87/NBC-650/21/62 in Pakistan, ensuring that all data collection adhered to local ethical standards, and informed consent was obtained from all caregivers.

### Measures

#### Early Learning (EL) Tool

The EL tool is an observational and caregiver-reported instrument consisting of 14 items. These items encompass play and stimulation activities that promote early child development (ECD) [9]. The EL tool development was commissioned in 2020 by the World Health Organization in order to create an evidence-informed measure for assessing early learning for monitoring and evaluation. The definition of early learning activities used was ‘stimulating engagement with objects (e.g. playthings) and/or people (adults and peers) where responsiveness may not occur’ [9]. Examples of playthings include things for moving around (balls, wheels, etc.), things for role playing and pretending, things to manipulate (blocks, sticks, etc.), things that produce sound, picture books, and things with different shapes and colours. Examples of stimulating activities include reading or looking at books together, taking children out to visit friends and family, telling stories or rhymes to the child, singing to the child, playing games with the child and talking to their child. Based on this definition, a conceptual framework was generated, items were piloted and iterative qualitative and quantitative testing was conducted. The final EL tool contains 14 items and represents stimulating engagement with playthings and people.

To utilise the EL tool, first, a data collector enters the household asks caregivers if their child has access to a variety of different playthings (i.e. things for role-playing/pretending, things that produce sound), including playthings used in solitary or collective play, and involving objects from any origin whether bought, found in the home, or handmade. If the caregiver has the item, the data collector asks for the caregiver to present the item to them. Upon viewing the items presented by the caregiver, enumerators used the tool to answer each question with a ‘yes’ or ‘no’, which was respectively coded as 1 or 0. The data collector then asks the caregivers whether they had participated in any stimulating activities with their child in the past 24 hours (i.e. Did you take your child out to visit friends, family, or to a shop? Did you tell stories or rhymes to the child?), and the data collector codes using the same methodology. The total possible score is a sum of the 14 yes/no items and could range from 0 to 14.

#### Evaluation of Home Environment-Infant Toddler Version (HOME-IT)

The HOME-IT was used as a criterion measure for the quality of stimulation within the home setting [7]. It has previously been used in Pakistan [12,19], and operates as an observational tool. Although authors argue that the HOME-IT is composed of six subscales (responsivity, acceptance, organisation, learning materials, involvement, and variety), prior research conducted in LMICs found these six subscales load on to a variety of factors, dependent on the context [13]. In our study setting, an exploratory factor analysis (EFA) revealed that the loaded onto a single factor, with a Cronbach’s alpha of 0.67. Therefore, we used the aggregated HOME score to quantify overall early stimulation.

#### Responsive Care (RC) Tool

The RC tool was used as a correlation measure, as we would expect a valid measure of early learning to positively correlate with responsive interactions. The RC instrument employs an observational method of a semi-structured interaction between caregiver and child to categorise caregiver responsive behaviours [9]. For this study, we used one dimension of caregiver behaviour: responsive interactions. This procedure entails giving caregivers a culturally and age-appropriate picture (for the verbal interaction task) or a toy (for the play task), prompting them to engage with their child in a typical manner. Concurrently, an observer tallies the caregiver's behaviours during two live observations: a five-minute verbal interaction and a five-minute play interaction. A total responsive interactions score is calculated by summing the number of responsive interactions over the five-minute interval. The validation of the RC tool, based on this sample, has been published elsewhere [17], showing evidence that the RC tool is highly reliable and valid in this population (Cronbach’s alpha of 0.93 for responsive interactions).

#### Sociodemographic survey

Caregivers filled out a sociodemographic survey that included information about the mother's and father's occupation and years of education, languages spoken, number of births (parity), and household asset ownership details (e.g. home ownership status, primary source of drinking water, ownership of cattle, buffalos, and cows, etc.). Principal component analysis was used to calculate an overall wealth score for each household [23]. This survey was previously administered in other studies in Naushahro Feroze [21].

#### Bayley Infant Development Scale, 4th edition

The BAYLEY-4 [24] was implemented to evaluate early childhood development (ECD), and to assess predictive validity, as we would expect higher levels of early learning to be positively associated with ECD. It was selected as the comparison metric in our study due to its acceptance as a ‘gold standard’ clinical assessment in the field of early childhood development, with strong reliability and validity. The BAYLEY-4 assesses three domains of ECD: cognitive, communication (expressive and receptive), and motor (gross and fine). Two additional domains, adaptive behaviour and social-emotional, were assessed through a parent-report subtest. While the BSID 3rd edition was previously validated in Pakistan [21,22], this was the first study to use the 4th edition. As such, item-level adaptations were made to ensure cultural relevance. For example, names were changed (i.e. changed from Lee to Laila and Jamie to Jamila), and pictures were changed (i.e. a boy in western pajamas was changed to be wearing typical Pakistani pajamas) to be culturally relevant. Each subscale's scores were computed using raw scores from the BSIV-IV manual, with age adjustments for each analysis. Raw scores were manually calculated following the procedure detailed in the BAYLEY-4 manual.

### Statistical analysis

Descriptive analyses were performed to examine the characteristics of our sample. To assess the construct validity of the EL tool, we tested the model fit of a one-factor solution, including all 14 items, and a two-factor solution, with stimulation with playthings (items 1–7) and stimulation with people (items 8–14) as the two factors of interest. In order to test these two factor structures, two confirmatory factor analyses (CFA), a statistical method used to assess the relationship between individual items and their underlying latent factor, were conducted on the full sample (n = 200) and a likelihood-ratio test was used to identify the better fitting model (one vs. two factors). After the better fitting model was selected, global fit statistics were computed to assess how well the model fit the data (Root Mean Square Error of Approximation (RMSEA)<0.08, Comparative Fit Index (CFI)>0.90, Standardized Root Mean Square Residual (SRMR)<0.08) [25]. If the fit statistics or factor loadings were inadequate (factor loading <0.32) [26,27], an Item Response Theory (IRT) graded response model (GRM) was used to identify the level of information contributed by each item across underlying levels of theta and the scale was refined accordingly. An IRT GRM was selected as it provides an understanding of individual item information (e.g. how ‘helpful’ an item is at describing the underlying latent construct), across varying levels of the underlying latent construct (e.g. an item might provide a lot of information for a household with low levels of underlying early learning but not a lot of information with households with high levels of underlying early learning).

Criterion validity was assessed by computing the correlations between the sum score of each of the identified EL subscales and the overall HOME sum score. We hypothesised that the EL sum score(s) would be positively and significantly correlated with the overall HOME sum score. The correlations between the EL sum score(s) and the score on the RC measure’s responsive interactions subscale, in addition to a set of family characteristics (e.g. maternal education, and household wealth) were also calculated. We hypothesised that all the EL subscale(s) would be positively and significantly correlated with responsive interactions, maternal education, and household wealth. Finally, we used a traditional ordinary least squares linear regression model to explore the EL tool’s predictive validity. Specifically, we conducted individual regression analyses where each of the identified subscale scores of the EL tool was used to predict the individual dimensions of ECD (cognitive, receptive communication, expressive communication, fine motor, gross motor, and social emotional dimensions) as measured by the BAYLEY-4 raw scores, controlling for child age. All data computations were performed using Stata v17. Study materials can be accessed online [9], and anonymised data and codes are available upon request.

## RESULTS

### Descriptive

Table S1 in the **Online Supplementary Document** reports the characteristics for the study sample. Fifty-two percent of our sample of mothers had no formal schooling, 28% completed some primary school, and 20% completed primary or above. Most mothers were housewives (96.5%), who lived with their extended family (72.5%), spoke a local language at home, including Sindhi, Siraiki or Balochi (72.5%), and had 1–2 children (54.0%).

Most mothers spoke with their child while they were busy with housework (94%), but very few children had picture books (6%) or things with different shapes and colours (2%). Satisfactory item-test correlations, ranging between 0.22 (things with different shapes and colours, or for drawing shapes, colours) and 0.65 (things for role-playing, pretending (e.g. dolls, household items, plane, cars), were found (**Table 1**).

**Table 1:** Descriptive statistics for Early Learning

Variable	x̄	SD	Min	Max
1. Things for moving around (balls, wheels, push & pull)	0.35	0.48	0	1
2. Things for role-playing, pretending (e.g. dolls, household items, plane, cars)	0.55	0.50	0	1
3. Things to manipulate: to fill, stack, construct, build (blocks, sticks, stones)	0.22	0.41	0	1
4. Things that produce sound (e.g. drum)	0.15	0.35	0	1
5. Child has picture book (not textbook, can be collection of pictures)	0.06	0.24	0	1
6. Things with different shapes and colours, or for drawing shapes, colours	0.02	0.12	0	1
7. Is there a designated and accessible place for child’s playthings?	0.12	0.32	0	1
8. In the past 24 h, did you read or look at pictures in a book, calendar, or magazine with the child?	0.24	0.43	0	1
9. In the last 24 h, did you take your child out to visit friends, family or to shop?	0.67	0.47	0	1
10. In the last 24 h, did you tell stories or rhymes to the child?	0.32	0.47	0	1
11. In the last 24 h, did you sing songs or lullabies with the child?	0.69	0.46	0	1
12. In the last 24 h, did your child play any structured games with people, like circle games, clapping, singing or ones with objects?	0.41	0.49	0	1
13. When you are busy with housework, in the last 24 h, did you talk with your child?	0.94	0.24	0	1
14. In the past week, did you give your child a new plaything? (what?)	0.26	0.44	0	1

Min – minimal, Max – maximal, SD – standard deviation, x̄ – mean

### Analysis 1. Construct validity

Two CFAs were conducted using data from the EL tool: one in which all items loaded onto a single factor and one in which items loaded onto two separate factors reflecting stimulation with playthings and stimulation with people. Results from a maximum likelihood-ratio test indicated that the two-factor solution was the better fit (*P* = 0.030). Fit statistics for the two-factor solution were as follows: *x^2^* (76) = 182.578, *P* < 0.001; RMSEA = 0.084; CFI = 0.782; SRMR = 0.076). For the two-factor solution, RMSEA and CFI fit statistics were unsatisfactory, and three items had factor loadings below the cut-off of 0.32: item 3 (‘things to manipulate’), item 6 (‘things with different shapes and colours’) and item 13 (‘when you are busy with housework, in the last 24 hours, did you talk with your child?’). One other item, item 1 (‘things for moving around’), loaded onto the first factor just above 0.32, at 0.35.

Given the theoretical importance of each item, an IRT graded response model was fit to the full scale and full sample to estimate item location and information parameters. Item information parameters ranged from 0.300 for Item 3 to 2.637 for item 7 (‘is there a designated and accessible place for child’s playthings?’) (Figure S1 in the **Online Supplementary Document**). Notably, although the average factor loading for item 13 (‘when you are busy with housework, in the last 24 hours, did you talk with your child?’) was below 0.3, this item provided a lot of information for individuals with low underlying early learning scores, or households with children who engaged in limited levels of stimulation with playthings and people. Similarly, although the average factor loading for item 6 (‘things with different shapes and colours, or for drawing shapes, colours’) was also below 0.3, it provided a lot of information for individuals with high underlying early learning scores, or households with children who engage in higher levels of stimulation with playthings and people. Items 1 and 3 provided very little information across all levels of early learning. Therefore, we hypothesised that the EL scale may function similarly without items 1 and 3. To test this hypothesis we constructed a test information function with the full scale (14 items) and the shortened scale (12 items) (Figure S2 in the **Online Supplementary Document**). Based on these results, the 12-item scale provides almost the same amount of information as the full 14-item scale. Further, the internal consistency reliability of the 12-item scale was almost identical to that of the 14-item scale, 0.774 vs. 0.769, respectively, which indicated that the 12-item scale was a reliable and precise estimate of early learning. The two factors were highly correlated at 0.94 once items one and three were dropped.

Therefore, we refit the structural equation model with two latent constructs without items 1 and 3. Fit statistics were as follows: *x^2^* (53) = 83.492, *P* = 0.005; RMSEA = 0.054; CFI = 0.923; SRMR = 0.055), indicating satisfactory fit (Figure S3 in the **Online Supplementary Document**).

### Analysis 2. Criterion validity

Sum scores for the two EL tool subscales were calculated from the five play-thing items and the seven stimulations with people items, respectively. Given the fact that most end users of this tool are expected to prefer simple sum scores over factor scores, we conducted our criterion and predictive validity analyses by correlating the sum scores by our variables of interest. In the criterion validity analysis, the EL tool stimulation with playthings subscale was strongly and positively associated with the overall HOME sum score (Pearson Correlation = 0.68, *P* < 0.01) and the EL tool stimulation with people subscale was strongly and positively correlated with the overall HOME sum-score (Pearson Correlation = 0.65, *P* < 0.01).

### Analysis 3. Correlations with responsive interactions, maternal education, and wealth

Both EL subscales (stimulation with playthings and stimulation with people) were positively and statistically significantly correlated with more responsive interactions, higher levels of maternal education, and higher levels of wealth (**Table 2**). Stimulation with playthings and people were both most strongly correlated with responsive interactions, with a higher correlation seen between stimulation with people and responsive interactions.

**Table 2:** Correlation coefficients between Early Learning variables and variables of interest*

Variables of interest	Stimulation with playthings	Stimulation with people
Responsive interactions	0.45	0.57
Maternal education	0.31	0.26
Wealth score	0.32	0.29

*Indicates significance at α = 0.01 for all values.

### Analysis 4. Predictive validity

Results of two separate ordinary least squares linear regression models showed that stimulation with playthings and stimulation with caregivers were both positively and statistically significantly associated with children’s cognitive development, receptive communication, fine motor skills and gross motor skills (**Table 3**). Neither dimension of EL was significantly associated with children’s expressive communication or social emotional skills. Stimulation with playthings was more strongly associated with children’s cognitive development, receptive communication, and expressive communication compared to stimulation with caregivers.

**Table 3:** Regression results predicting child development from Early Learning subscales, with 95% confidence intervals

Child Development Dimension	Cognitive development	Receptive communication	Expressive communication	Fine motor	Gross motor	Social emotional
Stimulation with playthings	5.36* (3.66, 7.04)	2.82* (1.75, 3.88)	0.71 (−0.46, 1.87)	2.34* (1.10, 3.58)	5.16* (3.28, 7.05)	2.79 (−0.12, 5.71)
Stimulation with caregivers	2.25* (1.34, 3.16)	1.31* (0.75, 1.87)	0.56 (−0.05, 1.16)	1.83* (1.22, 2.45)	2.40* (1.41, 3.40)	1.49 (−0.03, 3.00)

*Indicates significance at α = 0.01.

## DISCUSSION

In the present study the validity of a tool developed to measure young children’s early learning at home during a task was assessed in rural Sindh, Pakistan. Our findings indicated that the EL tool is valid and can be implemented in under 5-minute by trained data collectors. Evidence from the comparative CFA indicated that the tool captured two distinct dimensions of early learning, one representing stimulation with playthings (i.e. ‘things that produce sound’, ‘things with different shapes and colors’, ‘things to manipulate’) and one representing stimulation with people (i.e. ‘Did you tell stories or rhymes to your child?’, ‘Did you sing songs or lullabies with the child?’, ‘Did you talk with your child?’). An IRT analysis revealed that two items should be excluded from the tool in this sample due to low levels of information across theta, item one (‘things for moving around’), and item three (‘things to manipulate’).

Both subscales of the EL tool demonstrated positive correlations with a current commonly used measurement of early stimulation, the overall HOME-IT sum score, suggesting the tool’s criterion validity. Both subscales were positively correlated with higher levels of responsive interactions, maternal education, and wealth. Predictive validity was also demonstrated by a positive association between both dimensions of EL and all dimensions of ECD, except for social-emotional and expressive communication. Overall, given the ease of use and strong psychometric principles, the EL tool is a promising measure of early learning, as measured by the NCF, has proven to be valid in Naushahro Feroze and has the potential to be valid in other LMIC settings.

Despite strong evidence supporting two distinct subscales in the EL tool, it should be noted that the one-factor solution also provided adequate fit statistics. Furthermore, the two factors in the EL tool were highly correlated once items one and three were dropped. The two-factor solution without items one and three fit best in this context, but the two items that were dropped (‘things for moving around (balls, wheels, push & pull)’ and ‘things to manipulate: to fill, stack, construct, build (blocks, sticks, stones)’, respectively) are conceptually important to the underlying meaning of the EL tool, but did not provide adequate information about the underlying construct of stimulation with playthings in this context. Therefore, users interested in a single EL score, or those who also believe items one and three to be theoretically important to their context should consider including them and/or using an overall sum-score. In other settings, the first time this tool is used, a similar analysis should be run to understand the level of information provided from each individual item and a theoretical and statistical decision should be made based on that information.

Importantly, despite low levels of overall information provided by item six (‘things with different shapes and colors, or for drawing shapes, colors’) and item eleven (‘In the last 24 hours, did you sing songs or lullabies with the child?’), an IRT GRM allowed us to highlight the importance of each of these items in providing information about those with very low-levels and very high-levels of underlying early learning, for items eleven and six, respectively. This highlights the importance of investigating the underlying factor structure with IRT, as CFA only provides the average factor loading across theta.

Concurrent and predictive validity evidence points to the strong correlation between stimulation with playthings and people, and higher levels of maternal education, wealth, responsive interactions, and child development. Though responsive interactions and early learning are distinct concepts, they often occur simultaneously. Higher levels of household wealth have consistently been found to be associated with higher levels of early learning [28,29], and predictive validity findings are consistent with existing evidence that indicates that higher levels of stimulation improve child development [30,31]. Further, evidence from the FCI validation study in Bangladesh found that the play activities and varieties of play materials were most strongly correlated with child development [8]. It should be noted, however, that although both domains of early learning were positively correlated with all domains of child development, they were not significantly associated with expressive communication or social emotional skills. Although early learning is correlated with child development overall, most evidence focuses on cognitive and motor development [32,33]. Future analyses should assess the extent to which early learning predicts children’s social emotional development, as this pathway is much less understood.

Though we believe this study makes an important contribution to the literature, it should be noted that data were collected on all variables cross-sectionally. As such, the extent to which this study could assess the predictive validity of early learning on child development is limited, as reverse causation could be in play (i.e. children that are more developed are easier to interact with and causes parents to engage in more early learning activities with them). Follow-up data collection would help to identify the longitudinal impact of early learning on ECD over time. Additionally, we did not collect inter-rater reliability between data collectors or test-retest data over time, and the sample size was limited to 200 households. However, despite a limited sample of 200 caregiver-child dyads, the model sufficiently fit the data as indicated by the CFA and IRT analyses. Future work should assess the reliability of the tool and should replicate this study in different contexts, as the validity of the EL tool is specific to this context in rural, Sindh Pakistan and is not generalisable to other contexts.

The results of this study suggest that the EL tool can be used to determine levels of early learning. Specifically, the EL tool would be useful to implement as a pre-test and post-test measure for an intervention with the goal of improving early learning practices of caregivers in a given community. Due to the caregiver-reported nature of the tool, the EL tool also has the potential to be useful for larger scale intervention evaluations or a population-level survey, such as the Multiple Indicator Cluster Survey or Demographic and Health Survey. The tools’ explicit focus on the 0–3-year age group, the option to utilise a one-factor (measuring early learning) or two factor (measuring stimulation with playthings and stimulation with people) solution, and its’ validity in a low-income, low-resource setting provides a novel contribution to the literature.

## Additional material


Online Supplementary Document
