# Supplementary material for: The development and validity of the Early Learning Tool for children 0–3-year-old in rural Pakistan
Source: J Glob Health. 2024 Nov 22;14:04241. doi: 10.7189/jogh.14.04241 (PMC11586645; doi:10.7189/jogh.14.04241)

Table 1

Sample Characteristics, N=200

Socio-Demographic Characteristic	Count (n)	Percent (%)
Maternal Depressive Symptoms (SRQ-20>=9)	45	22.5
Maternal Occupation		
Housewife	193	96.5
Nurse, Teacher, Clerk, Handicraft, or Lady Health Worker	7	3.5
Family Structure		
Lives with extended family	145	72.5
Nuclear family	55	27.5
Primary Language Spoken		
Sindhi	145	72.5
Local Language (Sindhi, Siraiki or Balochi)	54	27.0
Urdu	1	0.5
Maternal Age		
(mean, sd)	28.6	6.4
<19	4	2.0
19-24	57	28.5
25-29	49	24.5
30-39	75	37.5
>= 40	15	7.5
Maternal Years of Schooling		
(mean, sd)	3.4	4.3
None	104	52.0
Some Primary School	56	28.0
Primary School or Above	40	20.0
Maternal Parity		
(mean, sd)	2.8	1.8
1 to 2	108	54.0
3 to 4	56	28.0
>= 5	36	18.0

Figure 1

IRT GRM Item Information Function for Early Learning Scale

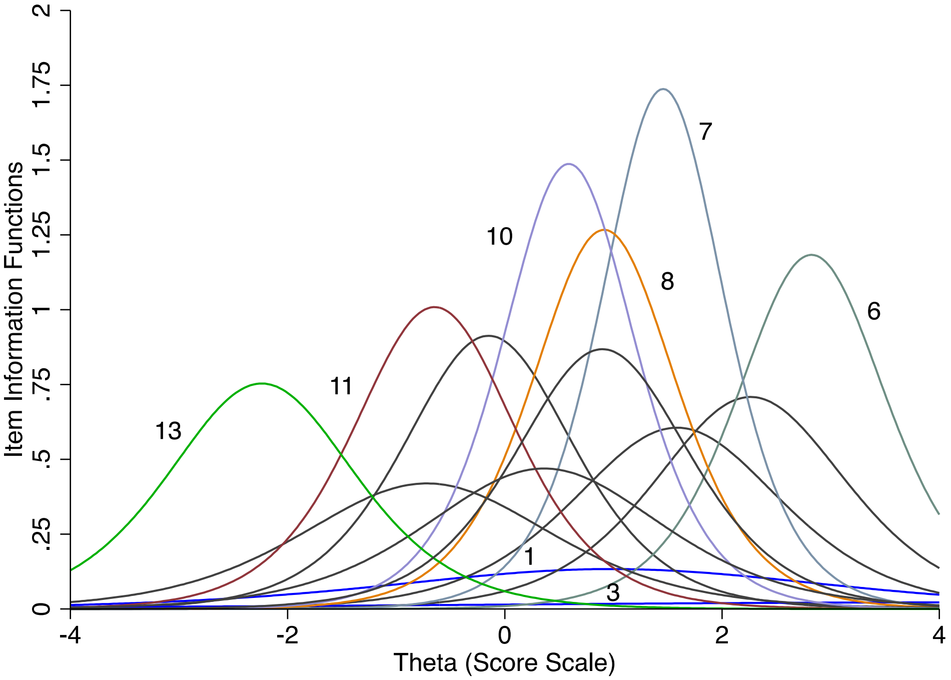

Figure 2

IRT GRM Test Information Function for 14-item and 12-item Early Learning Scales

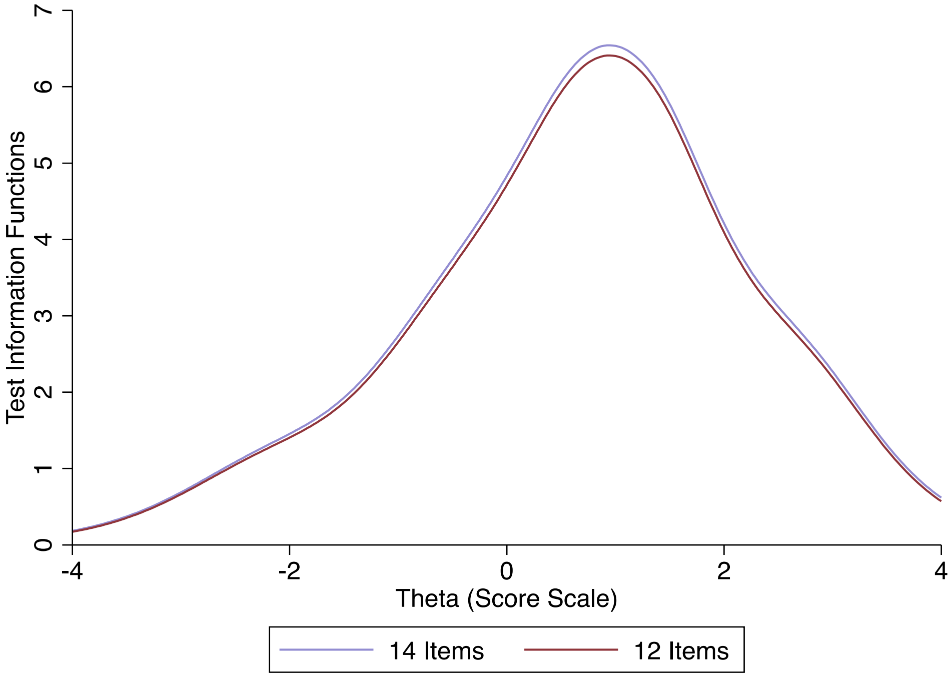

Figure 3

Measurement Model for Latent Constructs

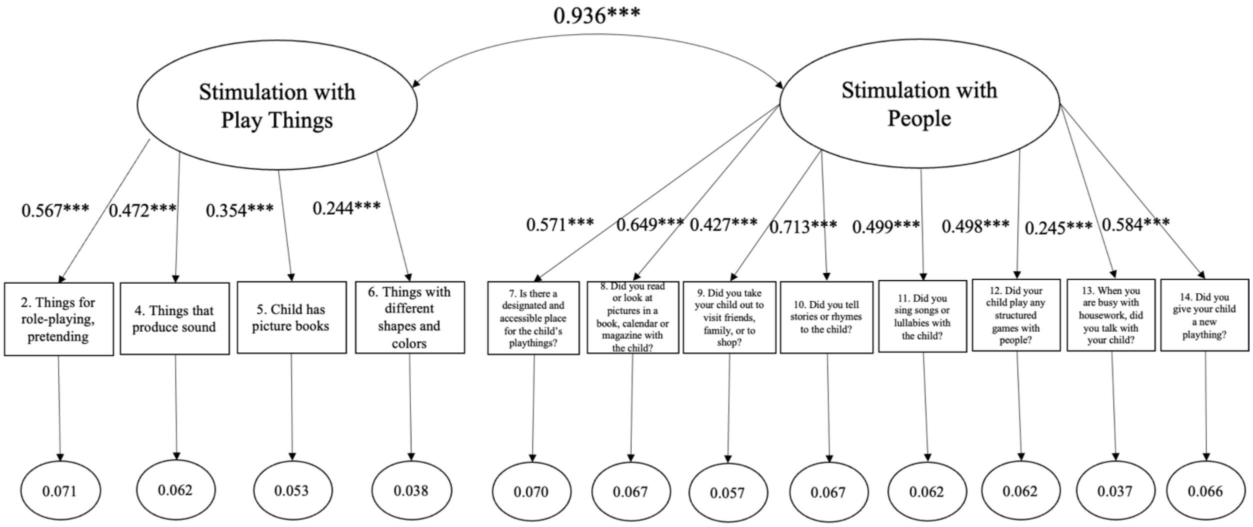

Supplement: Online Supplementary Document [file jogh-14-04241-s001.pdf]
